# PROFILE OF CAREGIVING ACTIVITIES AND ASSOCIATION WITH PHYSICAL HEALTH AMONG DEMENTIA SPOUSAL CAREGIVERS

**DOI:** 10.1093/geroni/igad104.2526

**Published:** 2023-12-21

**Authors:** Jinmyoung Cho, Laura Sands, Alan Stevens, Heather Allore, Molly Horstman, Rose Milius, Amber Hickey

**Affiliations:** Saint Louis University, St. Louis, Missouri, United States; Virginia Tech, Blacksburg, Virginia, United States; Baylor Scott and White Health, Dallas, Texas, United States; Yale University, New Haven, Connecticut, United States; Michael E. DeBakey VA Medical Center, Houston, Texas, United States; Baylor Scott & White Research Institute, Temple, Texas, United States; Baylor Scott & White Research Institute, Temple, Texas, United States

## Abstract

The daily care of a person living with Alzheimer’s disease and related dementia (ADRD) is associated with threats to the health and well-being of family caregivers. Although more is known about the relationship between caregiving and psychosocial distress, far less is known about the relationship between caregiving and physical health and health behaviors. This study aims to identify intensity of caregiving experience profiles and compare the profiles to multidimensional physical health indicators and health behaviors among spousal caregivers for persons with ADRD. Using data from 152 spousal caregivers aged 65 and over, the intensity of caregiving experience (ICE) was measured as the number and frequency of health- and medical-related helping activities for his/her care recipient. Multidimensional health indicators included fatigue, sleep disturbance, physical functioning, pain interference, and general health from self-reports, and the number of chronic conditions from the electronic health records. Health promotion behaviors were assessed as: health responsibility, physical activity, nutrition, interpersonal relations, and stress management. Cluster analysis was performed to determine the number of ICE profiles. T-tests and chi2 tests were to assess differences in health indicators and behaviors between clusters. We identified two distinct caregiving experience patterns: high-intensity (37.5%) and low-intensity (62.5%) caregiving. Caregivers from the high-intensity caregiving cluster reported feeling more tired (t=2.254, p< .05), perceiving more sleep disturbance (t=3.06, p< .001), and performing less physical activity (t=2.05, p< .05). Future studies are needed to develop effective interventions to reduce sleep disturbance and fatigue among family caregivers who are heavily involved in caregiving activities.

